# The iHOPE-20 study: Relationships between and prospective predictors of remission, clinical recovery, personal recovery and resilience 20 years on from a first episode psychosis

**DOI:** 10.1177/0004867419827648

**Published:** 2019-02-06

**Authors:** Donal O’Keeffe, Ailish Hannigan, Roisin Doyle, Anthony Kinsella, Ann Sheridan, Aine Kelly, Kevin Madigan, Elizabeth Lawlor, Mary Clarke

**Affiliations:** 1 DETECT Early Intervention in Psychosis Service, Dublin, Ireland; 2 School of Nursing and Midwifery, Trinity College, The University of Dublin, Dublin, Ireland; 3 Graduate Entry Medical School, Faculty of Education and Health Sciences, University of Limerick, Limerick, Ireland; 4 Department of Molecular and Cellular Therapeutics, Royal College of Surgeons in Ireland, Dublin, Ireland; 5 School of Nursing, Midwifery and Health Systems, University College Dublin, Dublin, Ireland; 6 Research Department, Saint John of God Hospitaller Services, Dublin, Ireland; 7 St. John of God Community Services, Dublin, Ireland; 8 School of Postgraduate Studies, Faculty of Medicine and Health Sciences, Royal College of Surgeons in Ireland, Dublin, Ireland; 9 School of Medicine, University College Dublin, Dublin, Ireland

**Keywords:** First episode psychosis, follow-up, recovery, resilience, iHOPE-20

## Abstract

**Objective:**

Knowledge of outcome in psychotic illness is limited by the paucity of very long-term epidemiologically representative studies of incidence first episode psychosis (FEP) cohorts that measure and compare outcomes reflecting modern clinical practice, mental health policy and research agendas. Our study aimed to address this gap.

**Method:**

iHOPE-20 is a prospective 20-year follow-up study of a FEP incidence cohort (*N* = 171) conducted between 2014 and 2017 in Ireland. Data from previous studies and medical records were used to recruit cohort members. We assessed remission, clinical recovery, personal recovery and resilience at 20 years; explored the relationships between these outcomes and examined the predictive value of baseline characteristics in determining them.

**Results:**

At follow-up, 20 out of 171 cohort members (11.70%) were deceased. We assessed 80 out of 151 alive cohort members (53% recruitment rate); 65% were in remission; 35.2% were in Full Functional Recovery and 53.7% confirmed they were fully recovered according to their personal definition of recovery. A complex array of relationships between outcomes was found. Outcomes were better for people who had a short duration of untreated psychosis, displayed higher premorbid social adjustment (between the ages of 5–11) and at baseline, were older, not living alone, in full-time employment, given a non-affective diagnosis, and had lower Global Assessment of Functioning scores.

**Conclusion:**

Among participants, full remission of psychotic symptoms and personally defined recovery was not just possible but likely in the very long term. However, attaining positive functional outcomes and building resilience in FEP remain key challenges for mental health services.

## Background

Since the World Health Organization’s ground-breaking, cross-cultural, International Study of Schizophrenia (Hopper et al., 2007), longitudinal research has challenged the association of psychotic illness with chronicity (Lally et al., 2017). Evidence for the effectiveness of early intervention services suggests that assuming psychosis leads to progressive deterioration and negative outcome is a ‘clinician’s illusion’ (Marwaha et al., 2016). Recovery optimism and heterogeneity in psychosis have become cornerstones of the recovery movement. However, little is known about the long-term outcome of psychosis (⩾8 years post first episode psychosis [FEP]), with most long-term research describing schizophrenia and non-affective psychosis samples.

Follow-up studies of FEP cohorts vary regarding inclusion criteria, follow-up period and how outcomes are conceptualised. While long-term (8–20 year) follow-up studies of FEP cohorts exist, there is a dearth of very long-term FEP data (⩾20 years). Although studies have been conducted with recent onset, prevalence, first admission psychosis and first episode schizophrenia cohorts (Harrison et al., 2001; Kotov et al., 2017; Rangaswamy, 2012), this research may not fully account for the diversity of psychotic illness. These samples have been described as enriched, not representing the modern spectrum diagnostic approach (Guloksuz and Van Os, 2018). The selection of poorer outcome service users and the effect of relapses, psychiatric medication and illness duration can introduce bias which impacts generalizability (Conus et al., 2007). Longitudinal outcome research failing to include a wider taxonomy of psychosis may angle our understanding of its impact towards long-term impairment. Epidemiological evidence indicates that schizophrenia and bipolar disorder do not represent exclusive diagnostic entities (Baldwin et al., 2005). Prospective studies utilising incidence FEP cohorts recognise the considerable overlap between affective and non-affective psychosis (Tamminga et al., 2013) and provide the most accurate estimate of outcome (Simonsen et al., 2007).

Despite epidemiologically robust studies, data on outcome following a FEP are limited by the scarcity of very long-term follow-up designs and the restricted nature of measures utilised (Morgan et al., 2014). Contemporary mental health policy/services grounded in the recovery-oriented approach promote the appraisal of outcomes prioritised by service users. These extend beyond symptomatology and functioning to include personal (i.e. service user defined) recovery outcomes. Moreover, in the shift from illness-oriented to health-oriented services, there is a need to appraise positive outcomes in mental health – such as resilience (Thornicroft and Slade, 2014).

### Duration of untreated psychosis, duration of untreated illness and outcome

Seven independent systematic reviews have confirmed the strength of duration of untreated psychosis (DUP) in predicting clinical outcomes (i.e. positive symptoms, negative symptoms, relapse rates) (Penttilä, 2013). However, it is unknown if this link is maintained in the long term in all cases of psychosis. The predictive power of DUP may wane over time due to the masking effects of other variables (e.g. premorbid adjustment) and the cumulative effect of exposure to psychosis. Some studies have demonstrated how a longer duration of untreated illness (DUI: DUP + prodrome) predicted poorer outcome at follow-up (Bhullar et al., 2018; Crumlish et al., 2009; Norman et al., 2012); others have not found this effect (Barnes et al., 2008; González-Blanch et al., 2008; Ho et al., 2000). Determining whether DUP or DUI is a stronger predictor of long-term outcome necessitates comprehensive outcome assessment as the relationship appears to depend on the outcome variable measured.

To summarise, knowledge of outcome in psychotic illness is limited by the paucity of very long-term epidemiologically representative studies of FEP incidence cohorts that measure and compare outcomes reflecting modern clinical practice, mental health policy and research agendas. To our knowledge, no study has traced a FEP incidence cohort 20 years or more after initial diagnosis.

### Irish health outcomes in psychosis evaluation − 20-year follow-up study

To address these challenges, we carried out a prospective 20-year follow-up of a FEP incidence cohort (*N* = 171), between 2014 and 2017, in Ireland (Irish health outcomes in psychosis evaluation − 20-year follow-up study [iHOPE-20]). In this article, we report on the methodology of iHOPE-20’s quantitative component and examine, at 20 years: (1) the degree of remission, clinical recovery, personal recovery and resilience among the cohort; (2) the relationships between these outcomes and (3) the predictive value of baseline characteristics in determining them. iHOPE-20’s qualitative component has been reported on elsewhere (O’Keeffe et al., 2018). This involved purposefully sampling cohort members across the clinical recovery continuum to explore their experiences of mental health service use and recommendations for change 20 years after their FEP.

## Method

### Design and participants

The cohort researched is an epidemiologically complete FEP sample (*N* = 171), who represent all FEP service users referred to a private/public healthcare organisation, based in a Dublin catchment area where they resided. This is an urban region with a baseline population of approximately 165,000. At time of first contact (between February 1995 and February 1999), all referrals to inpatient and outpatient services in this catchment were screened by a team of psychiatrists. Individuals were included if they were aged ⩾12 and diagnosed with a FEP using the SCID-IV (structured clinical interview for DSM-IV axis I disorders) (First et al., 1995). FEP was defined as first presentation with acute psychotic symptoms to any psychiatric service for service users who, if they had been prescribed antipsychotic medication prior to presentation, had been receiving such treatment for not more than 30 days. Participants also met DSM-IV criteria for schizophrenia; schizophreniform disorder; delusional disorder; bipolar disorder; major depressive disorder with a first episode of psychotic features; substance-induced psychosis; psychosis due to a general medical condition and psychosis not otherwise specified. No cases of schizoaffective disorder or brief psychotic disorder were identified.

### Ethics, data protection and service user involvement

Ethical approval for the study was obtained from the Saint John of God Hospitaller Ministries Research Ethics Committee. In our reporting, we adhered to the strengthening the reporting of observational studies in epidemiology (STROBE) guidelines for cohort studies. All participants included in the 20-year follow-up were either deceased or provided written informed consent to complete an in-person assessment, a telephone assessment or a medical records access assessment. A service user contributed to study design and the selection of outcome measures.

### Tracing and recruitment procedures

Data from previous studies and medical records were used to recruit cohort members. We began by checking death records in the Irish General Registry Office and death notices online and ceased tracing anyone we identified as deceased. First contact was made by (1) requesting clinical teams/GPs to contact people by phone/in person at their next appointment or (2) by posted registered letters to cohort members’ last confirmed address. If the person expressed an interest in the study, they were invited by phone to meet us to learn about and discuss participation.

### Assessment instruments at baseline (1995–1999)

At first presentation, symptomatology was assessed using the Positive and Negative Syndrome Scale (PANSS) (Kay et al., 1987). Following a period of stabilisation (up to 72 hours), socio-demographics (age, gender, years in education, employment status, living alone status) were collected; each cohort member was diagnosed by a psychiatrist using the SCID-IV and the Global Assessment of Functioning (GAF) was administered (First et al., 1995). We also requested and obtained consent from each participant to interview their family. The Beiser Scale (Beiser et al., 1993) was completed separately with families and participants to measure the time (in months) between first presentation to psychiatric services for the initiation of treatment for psychotic illness and (1) the time of first onset of psychotic symptoms (DUP) and (2) the onset of prodromal symptoms (DUI). If an inconsistency was found between accounts, or if participants did not consent to a family interview, a consensus was obtained by utilising all information resources accessible. The Premorbid Social Adjustment Scale (Foerster et al., 1991) was used to determine participants’ social functioning during two discrete epochs in early life: age 5–11 (PSA1) and age 12–16 (PSA2). As the time period of PSA2 can often coincide with the onset of prodrome or psychosis, we utilised only PSA1 in our analysis. If family interview consent was not given by the participant, their PSA1 data were recorded as missing.

### Assessment instruments at 20-year follow-up (2014–2017)

Mean duration from baseline to follow-up was 19.68 years (SD = 0.82). At 20-year follow-up, we assessed remission by re-administering the PANSS (Kay et al., 1987). Excluding the 6-month duration component, we used Andreasen et al.’s (2005) remission criteria. Remission of positive and negative symptoms was defined as a score of ⩽3 on 8 PANSS items: delusions, unusual thought content, hallucinatory behaviour, conceptual disorganisation, mannerisms/posturing, blunted affect, social withdrawal and lack of spontaneity. We classified clinical recovery as ‘Full Functional Recovery’ (FFR) (Alvarez-Jimenez et al., 2012); a construct defined by combining remission status (from PANSS scores) with functional and vocational recovery status evaluated by the Quality of Life Scale (QLS) (Heinrichs et al., 1984). Functional and vocational status recovery was defined as a score of ⩾4 on 4 QLS questions: appropriate interpersonal relationships with people outside of family; adequate vocational functioning (being in paid employment, attending school or performing homemaker role effectively); adequate achievement in role adopted and basic living task engagement. Personal recovery was assessed in two ways. First (as a categorical variable), we asked participants a Personal Recovery Question (PRQ): ‘From your own understanding of what recovery is, do you consider yourself recovered?’. Response options were as follows: ‘Yes, fully recovered’; ‘Yes, partially recovered’ or ‘No, not recovered’. Second (as a point on a continuum), we administered the 24-item version of the Recovery Assessment Scale (RAS) (Corrigan et al., 2004). Resilience was measured using the Connor–Davidson Resilience Scale (CD-RISC) (Connor and Davidson, 2003). Some authors have theorised resilience as the process of individual effort to utilise resources deemed meaningful for wellbeing in the presence of adversity, which is supported by environmental conditions (Lal et al., 2017). However, in this study, we conceptualised resilience as an individual level outcome defined by personal qualities that enable thriving in the context of life challenges (Connor and Davidson, 2003).

### Statistical analysis

Numeric variables were tested for normality and summarised using mean and standard deviation (SD) for normally distributed variables and median and interquartile range (IQR) for skewed distributions. Chi-square (χ^2^) tests were used to test the association between categorical variables at baseline and group (assessed, not assessed/deceased). Independent samples *t* tests or Mann–Whitney tests were used to compare means or medians for baseline characteristics across group (assessed, not assessed/deceased). Pearson’s correlation coefficient was used to measure the strength of the association between DUP, DUI, CD-RISC and RAS scores. One-way analysis of variance was used to compare mean RAS and CD-RISC scores by personal recovery group (no, partial, full) with a Bonferroni post hoc test used for pairwise comparisons. Independent samples *t*-tests were used to compare mean RAS and CD-RISC scores between FFR groups. Kappa statistic was used to measure agreement between categorical outcomes. Linear regression was used to predict CD-RISC and RAS scores using the baseline predictors: age, sex, living alone (yes, no), in full-time employment (yes, no), premorbid social adjustment score, DUP/DUI in months, diagnosis (affective or non-affective), GAF score, PANSS total score, lifetime history of alcohol misuse or dependency (yes, no), and lifetime history of substance misuse or dependency (yes, no). The variance inflation factor was used to assess multicollinearity. *R* squared was used as a measure of goodness of fit and backward variable selection was used using the smallest partial correlation with the outcome as the criterion for removal. Logistic regression was used to predict binary outcomes of personal recovery (full, no/partial), FFR (yes, no) and PANSS remission (yes, no) using the same baseline predictors. Odds ratios with associated 95% confidence intervals are reported. The *c* statistic was used as a measure of predictive accuracy of the model. A sensitivity analysis was carried out to estimate remission/recovery rates for the full cohort using different outcome assumptions for those not followed up and those that died. IBM SPSS Statistics Version 23 and SAS Version 9.4 for Windows were utilised for analysis.

## Results

### Status at 20-year follow-up

**Figure 1.**
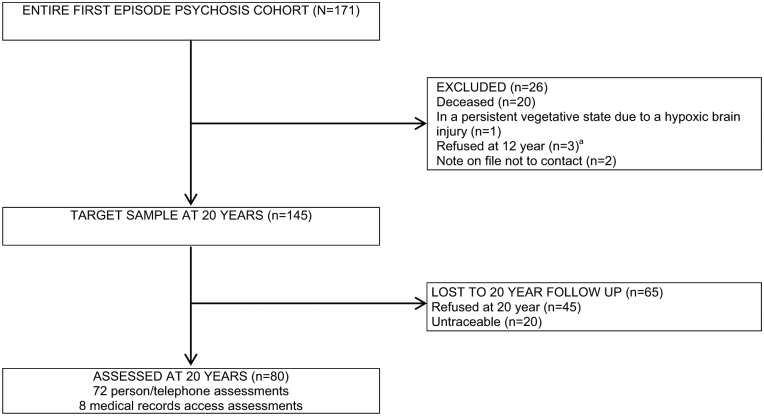
Flow chart of cohort member recruitment at 20 years. ^a^Ethical approval was contingent on us not attempting to contact cohort members who refused participation at 12-year follow-up.

At 20-year follow-up, 26 out of 171 participants were excluded from recruitment. Figure 1 displays the process of cohort member recruitment at 20 years and the reasons for these exclusions. This left a target sample of 145; of which, 48 out of 145 refused participation and 20 out of 145 were untraceable. We assessed 80 out of 151 living cohort members, giving us a recruitment rate of 53%. Eight of these 80 consented to medical records access only; for this group, we collected PANSS data from clinical notes. We confirmed 51 out of 80 were in contact with mental health services at assessment time. Of the 20 deaths, nine were due to natural causes, seven were due to unnatural causes and four had unknown cause of death.

### Differences between baseline characteristics of cohort members assessed and not assessed/deceased at 20-year follow-up

Baseline characteristics (age, gender, premorbid social adjustment, DUP, diagnosis, symptomatology, global functioning, employment status, substance abuse/dependence, alcohol abuse/dependence and living alone status) were compared across two groups (assessed, not assessed/deceased). Results presented in Table 1 demonstrate that no statistically significant differences in these baseline variables were found.

**Table 1.** Comparisons between baseline characteristics of cohort members assessed at 20-year follow-up (*n* = 80) and those not assessed/deceased (*n* = 91).

Baseline variable	Assessed (*n* = 80) *n* (%)/mean (SD)/median (IQR)	Not assessed/deceased (*n* = 91) *n* (%)/mean (SD)/median (IQR)	Total sample (*N* = 171) *n* (%)/mean (SD)/median (IQR)	Test statistic (*p*-value)
Age (years)^a^	24 (20.25–30)	26 (21–38)	25 (21–34)	*z* = –1.866 *p = *0.06^b^
Gender				χ^2^(1) = 2.114
Male	51 (63.75%)	48 (52.75%)	99 (57.9%)	*p = *0.15^c^
Female	29 (36.25%)	43 (47.25%)	72 (42.1%)	
Premorbid social adjustment (age 5–11)^a,d,e^	10 (8–13.25)	10 (8–13.25)	10 (8–13)	*z* = –0.161 *p = *0.87^b^
DUP (months)^a^	4 (1–22)	6 (1–24)	5(1–24)	*z* = –1.108 *p = *0.27^b^
Diagnosis				χ^2^(4) = 3.724
Schizophrenia spectrum disorder	56 (70%)	59 (67%)	115 (68.5%)	*p = *0.45^c^
Affective disorder	17 (21.3%)	17 (19.3%)	34 (20.2%)	
Substance-induced psychotic disorder	4 (5%)	7 (8%)	11 (6.5%)	
Psychotic disorder not otherwise specified	0 (0%)	2 (2.3%)	5 (3%)	
Organic		3 (3.4%)	3 (1.8%)	
Missing data		3	3	
PANSS total	75.63 (18.93)	70.47 (20.31)	72.86 (19.79)	*t*(166) = –1.695 *p* = 0.09^f^
GAF	24.08 (8.42)	21.89 (8.41)	22.91 (8.46)	*t*(168) = –1.689 *p* = 0.09^f^
Employment status				χ^2^(1) = 0.022
Full-time	29 (36.25%)	32 (35.16%)	61 (35.7%)	*p* = 0.88^c^
Not in full-time	51 (63.75%)	59 (64.84%)	110 (64.3%)	
Lifetime substance abuse/dependence				χ^2^(1) = 0.044
Yes	29 (36.71%)	32 (35.16%)	61 (35.9%)	*p* = 0.83^c^
No	50 (63.29%)	59 (64.84%)	109 (61.1%)	
Missing data	1		1	
Lifetime alcohol abuse/dependence				χ^2^(1) = 0.017
Yes	18 (22.50%)	21 (23.33%)	39 (22.9%)	*p *= 0.90^c^
No	62 (77.50%)	69 (76.67%)	131 (77.11)	
Missing data		1	1	
Living alone				χ^2^(1) = 1.824
Yes	10 (12.66%)	6 (6.59%)	16 (9.4%)	*p* = 0.18^c^
No	69 (87.34%)	85 (93.42%)	154 (90.6%)	
Missing data	1		1	

SD: standard deviation; IQR: interquartile range; DUP: duration of untreated psychosis; PANSS: Positive and Negative Syndrome Scale; GAF: Global Assessment of Functioning.

a Results presented as median (first quartile–third quartile).

b Mann–Whitney tests.

c Chi-square (χ^2^) tests.

d Missing data for premorbid social adjustment (age 5–11) for *n* = 43 cases.

e High premorbid social adjustment (age 5–11) scores indicate poor adjustment.

f Independent samples *t* tests.

### Assessment of outcomes

Table 2 summarises the five outcomes. PANSS remission was assessed in all 80 cases; 52 (65%) were deemed to be in remission. FFR was assessed in 71 cases; 25 (35.2%) were determined to be in FFR. All cases exhibiting QLS functional and vocational recovery were in remission. An answer to the PRQ was recorded for 67 cases; 36 (53.7%) described themselves as fully recovered. Four people objected to the PRQ stating that ‘Recovery is not an outcome’; ‘Recovery is a journey’; ‘I do not agree with the concept of recovery’ or ‘I am not in recovery as I was never unwell in the first place’. The mean RAS score was 97.72 (SD = 11.89) and the mean CD-RISC score was 66.89 (SD = 13.17). Of the 66 cases with information on all three categorical classifications of recovery, 23 (34.8%) were deemed to have met criteria for FFR; 36 (54.5%) considered themselves fully recovered according to the PRQ and 44 (66.7%) were in PANSS remission.

**Table 2.** Summary of remission, clinical recovery, personal recovery and resilience data and PANSS, QLS and GAF scores at 20-year follow-up (*n* = 80).

Measure	*n* (%)/mean (SD)
PANSS remission (*n* = 80)	
Yes	52 (65%)
No	28
QLS functional and vocational recovery (*n* = 71)	
Yes	25 (35.2%)
No	46
Missing data (*n* = 9)	
Declined to be interviewed	8
Did not complete interview	1
FFR (*n* = 71)	
Yes	25 (35.2%)
No	46
Missing data (*n* = 9)	
Declined to be interviewed	8
Did not complete interview	1
PRQ (*n* = 67)	
Full	36 (53.7%)
Partial	22 (32.8%)
No	9 (13.4%)
Missing data (*n* = 13)	
Declined to be interviewed	8
Objected to question	4
Did not answer question	1
RAS^a^ (*n* = 60)	97.7 (11.86)
Missing data (*n* = 20)	
Declined to be interviewed	8
Missing scale responses	12
CD-RISC^a^ (*n* = 64)	66.9 (13.17)
Missing data (*n* = 16)	
Declined to be interviewed	8
Missing scale responses	7
Did not complete interview	1
PANSS total (*n* = 80)	45.09 (13.03)
QLS total (*n* = 55)	91.16 (25.72)
Missing data (*n* = 25)	
Declined to be interviewed	8
Missing scale responses	16
Did not complete interview	1
GAF (*n* = 72)	65.97 (19.87)
Missing data (*n* = 8)	
Declined to be interviewed	8

GAF: Global Assessment of Functioning; SD: standard deviation; PANSS: Positive and Negative Syndrome Scale; QLS: Quality of Life Scale; FFR: Full Functional Recovery; PRQ: Personal Recovery Question; RAS: Recovery Assessment Scale; CD-RISC: Connor–Davidson Resilience Scale; GAF: Global Assessment of Functioning.

a High scores indicate high levels of personal recovery/resilience.

### Relationships between outcomes

The agreement between the classifications of remission/recovery is given in Table 3. The agreement between PANSS remission and PRQ response was moderate (kappa = 0.44) and higher than the agreement between FFR status and PRQ response (kappa = 0.32). The agreement between PANSS remission and FFR was moderate (kappa = 0.42). There was a strong positive correlation between scores for RAS and CD-RISC (*r* = 0.73, *p* < 0.001).

**Table 3.** PANSS remission and FFR status by PRQ categories (*n* = 66).

	PRQ (Fully Recovered [*n* = 36])
PANSS remission	
Yes	31 (86.1%)
No	5 (13.9%)
FFR status	
Yes	18 (50%)
No	18 (50%)
	PRQ (Not Recovered/Partially Recovered [*n* = 30])
PANSS remission	
Yes	13 (43.3%)
No	17 (56.7%)
FFR status	
Yes	5 (16.7%)
No	25 (83.3%)

PANSS: Positive and Negative Syndrome Scale; FFR: Full Functional Recovery; PRQ: Personal Recovery Question.

**Figure 2.**
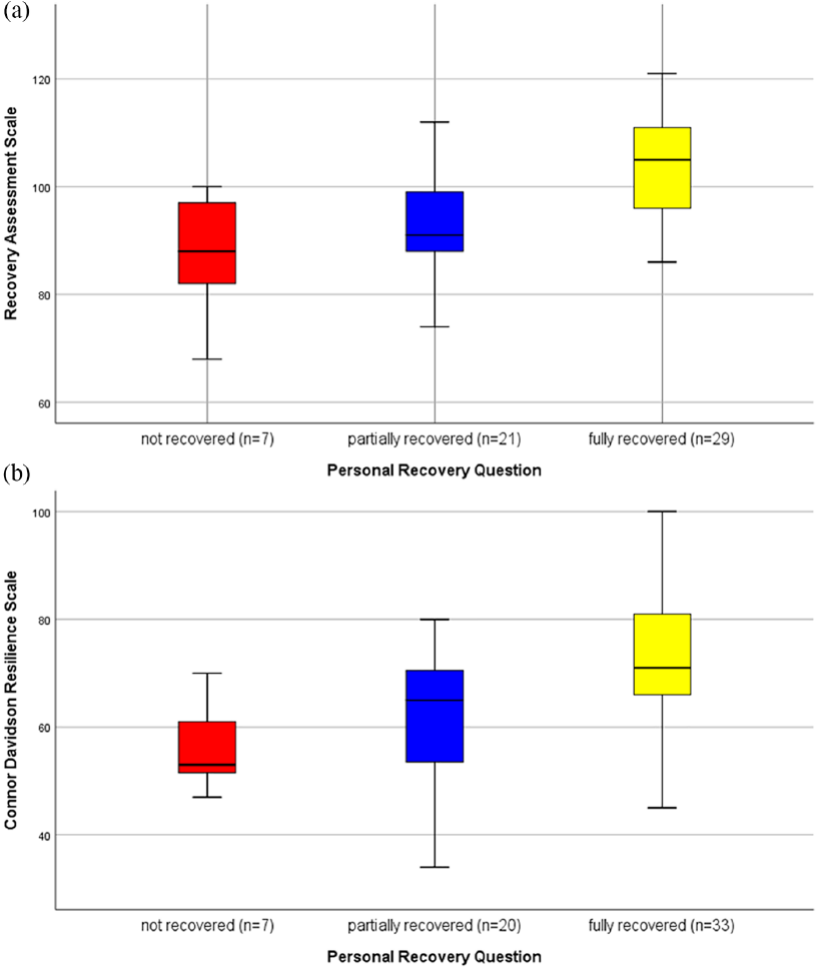
Box plots of RAS and CD-RISC scores by PRQ (no, partial, full). Missing data for some participants on each variable. For those who provided RAS data (*n* = 60); *n* = 3 were missing PRQ data. For those who provided CDRS data (*n* = 64); *n* = 4 were missing PRQ data.

Figure 2 summarises the distribution of scores for the RAS and CD-RISC by PRQ response (full, partial, no). There was a statistically significant difference in mean RAS scores across the three groups (*p* < 0.001) with pairwise comparisons showing the difference to be between PRQ Fully Recovered (RAS M = 103) compared to PRQ Not Recovered and PRQ Partially Recovered (RAS M = 88 and 93, respectively). Similarly, there was a statistically significant difference in mean CD-RISC across the three PRQ groups (*p* = 0.001) with pairwise comparisons showing the difference to be between PRQ Fully Recovered (CD-RISC M = 72) compared to PRQ (Not Recovered and PRQ Partially Recovered (CD-RISC M = 56 and 62, respectively). A statistically significant difference was found between mean RAS scores of participants in FFR (M = 103.3, SD = 8.67) and those who were not (M = 95.0, SD = 12.34); *t*(57) = –2.58, *p* = 0.013. Mean CD-RISC scores were similar across FFR groups (M = 68.7 for FFR compared to M = 66.0 for not FFR, *p* = 0.44).

### Baseline predictors of outcomes

Table 4 illustrates results of linear regression conducted to assess the ability of baseline variables to predict CD-RISC and RAS scores. DUP was a stronger predictor than DUI, explaining a higher percentage of variation in both outcomes so it was used instead of DUI in all models. DUP was a statistically significant predictor of both CD-RISC and RAS at 20 years (*p* < 0.05, Table 4) with longer DUP associated with lower scores on both scales at 20 years. Older age was associated with higher scores of CD-RISC and a primary diagnosis of an affective disorder was associated with lower RAS scores at 20 years. The percentage of variation explained by the model fitted for each outcome was low (adjusted *R* squared = 8% [CD-RISC] and 26% [RAS]).

**Table 4.** Linear regression of CD-RISC (*n* = 60) and RAS (*n* = 64) scores with baseline predictors.

Baseline variable	CD-RISC score at 20 years		RAS score at 20 years	
	β (95% CI)	*p*-value	β (95% CI)	*p*-value
Age (years)	0.41 (0.03, 0.80)	**0.04** *		
DUP (months)	−0.19 (–0.37, –0.02)	**0.03** *	−0.15 (–0.30, –0.01)	**0.04** *
In full-time employment	−4.50 (–11.52, 2.51)	0.20		
Primary diagnosis (affective)			−7.68 (–15.33, –0.03)	**0.04** *
Lifetime substance misuse or dependency			−4.22 (–10.50, 2.07)	0.18
Adjusted *R* squared	8%		26%	

CD-RISC: Connor–Davidson Resilience Scale; RAS: Recovery Assessment Scale; CI: confidence interval; DUP: duration of untreated psychosis.

* *p* < 0.05. * values are bolded.

Table 5 summarises the results of the logistic regression to predict FFR, PANSS remission and personal recovery. Premorbid social adjustment was a statistically significant predictor of all three measures of recovery (*p* < 0.05) with higher PSA1 scores at baseline (indicating poorer premorbid social adjustment) associated with reduced odds of recovery outcomes at 20 years. Being in full-time employment and not living alone at baseline were associated with increased odds of FFR. The values of the *c* statistic ranged from 0.73 (PRQ) to 0.87 (FFR).

**Table 5.** Logistic regression of FFR, PANSS remission and PRQ (Fully Recovered) (*n* = 66).

Baseline variable	FFR		PANSS remission		PRQ (Fully Recovered)	
	OR (95% CI)	*p*-value	OR (95% CI)	*p*-value	OR (95% CI)	*p*-value
Full-time employment						
Yes	9.30 (1.90, 45.56)	**0.006** *				
No	Reference					
Premorbid social adjustment (age 5–11)	0.78 (0.63, 0.97)	**0.02** *	0.81 (0.69, 0.95)	**0.01** *	0.84 (0.72, 0.98)	**0.02** *
Primary diagnosis						
Affective	6.68 (0.86, 52.06)	0.07				
Non-affective	Reference					
GAF	0.90 (0.81, 0.99)	**0.046** *				
Living alone						
No	17.82 (1.09, 291.05)	**0.043** *	4.62 (0.40, 53.65)	0.22		
Yes	Reference		Reference			
Lifetime use of alcohol						
Yes	3.51 (0.57, 21.76)	0.18				
No	Reference					
DUP			0.97 (0.93, 1.033)	0.07	0.98 (0.94, 1.01)	0.16
*c* statistic	0.87		0.79		0.73	

FFR: Full Functional Recovery; PANSS: Positive and Negative Syndrome Scale; PRQ: Personal Recovery Question; OR: odds ratio; CI: confidence interval; GAF: Global Assessment of Functioning; DUP: duration of untreated psychosis.

* *p* < 0.05. * values are bolded.

Table 6 estimates the PANSS remission, FFR and personal recovery rates for the full cohort (*n* = 171) using different outcome assumptions for those not followed up and for those that died. Assuming the recovery rate in those not followed is the same as those followed up and assuming all deaths did not recover results in a full cohort PANSS remission rate of 57%, FFR rate of 31% and personal recovery rate of 47%.

**Table 6.** Estimated remission and recovery rates for the full cohort (*n* = 171) using different outcome assumptions for those not followed up and for those that died.

Assumption for those not followed up	Assumption for those that died (*n* = 20)	Estimated PANSS remission rate (*n* = 171)^a^	Estimated FFR rate (*n* = 171)^b^	Estimated full personal recovery rate (*n* = 171)^c^
None recovered	All deaths not recovered	52 (30%)	25 (15%)	36 (21%)
None recovered	All deaths due to unnatural causes (*n* = 7) not recovered	65 (38%)	38 (22%)	49 (29%)
Recovery rate the same as in cohort followed up	All deaths not recovered	98 (57%)	53 (31%)	81 (47%)
Recovery rate the same as in cohort followed up	All deaths due to unnatural causes (*n* = 7) not recovered	111 (65%)	66 (39%)	94 (55%)
All recovered	All deaths not recovered	123 (72%)	105 (61%)	120 (70%)
All recovered	All deaths due to unnatural causes (*n* = 7) not recovered	136 (80%)	118 (69%)	133 (78%)

PANSS: Positive and Negative Syndrome Scale; FFR: Full Functional Recovery; PR: personal recovery.

a PANSS remission assessed in 80 with a remission rate of 65%.

b FFR assessed in 71 with a recovery rate of 35.2%.

c PR assessed in 67 with a recovery rate of 53.7%.

## Discussion

Our study presents unique data on very long-term outcome for an incidence FEP cohort. The distinctive sample utilised and the recovery-oriented approach to outcome assessment adopted, allowed for an evaluation of the level of agreement between clinical and personal conceptualisations of recovery as well as resilience at 20 years. Sixty-five percent of participants were in PANSS remission, 35.2% were in FFR and 53.7% confirmed they were fully recovered according to their personal definition of recovery. Results are similar to the WHO International Pilot Study of Schizophrenia prevalence cohort 25-year follow-up that found 60.2% of cohort members to be practically symptom free or with non-disabling residual symptoms (Harrison et al., 2001). Findings contrast with a recent 20-year first admission psychosis follow-up that found intensified symptom burden over time (Kotov et al., 2017). Although our FEP sample and measures utilised are not directly comparable to the meta-analytic clinical recovery evidence in schizophrenia (Hegarty et al., 1994; Jääskeläinen et al., 2013), our data nonetheless challenge the poor outcomes reported in this literature. However, the mean resilience score of our participants was markedly less than general population norms (participants: 66.9; population norm: 80.7) (Connor and Davidson, 2003). When comparing our findings to other studies, our low baseline GAF scores should be taken into account.

To our knowledge, the only other study that has utilised the same FFR criteria with a FEP sample found a lower rate of FFR at 7.5 years (26%) (Alvarez-Jimenez et al., 2012). By applying these strict criteria, our findings suggest that there are limited gains to be made in clinical recovery over the long term. This corresponds with the results of a meta-analysis which questioned the effectiveness of mental health services in enhancing long-term social, occupational and educational functioning in FEP (Lally et al., 2017). However, it is short-sighted to assume that participants did not fare well at 20 years, as over half of our sample concluded they were fully recovered themselves. A key finding was the discrimination between RAS and CD-RISC scores using participants’ own definition of recovery. We found substantial agreement between participants identifying as fully recovered (PRQ) and if they were in remission. There was also considerable concordance between participants classifying themselves as not recovered/partially recovered (PRQ) and if they were not in FFR. Results support the construct validity of the RAS, point to the role of symptomatology and functioning in personal recovery, and (when considered in combination with the correlation found between RAS and CD-RISC scores) underscore the congruence between personal recovery and resilience.

Presented data detail a complex interwoven relationship between personal and clinical recovery. Results suggest that they are not discrete constructs but also not significantly related. This is in line with a meta-analysis of the association which concluded that symptom severity only partially explains personal recovery (Van Eck et al., 2017). We found that some people do not identify with the concept of recovery but are happy to discuss symptoms and functioning. There is a risk with the international adoption of the recovery-oriented approach that aspects of recovery prioritised by service users (i.e. symptoms and functioning) become overshadowed by the emphasis on recovery principles. This may inadvertently marginalise people who cannot relate to the term ‘recovery’, which is counterproductive as the recovery movement the seeks to integrate all service user perspectives to optimise service delivery. Findings also indicate that efforts to increase resilience, in mental healthcare, could impact personal and clinical recovery. While the association we found may be explained by psychotic episodes depleting resilience; this relationship might also be mediated by the positive impact of exposure to adversity and the experience of psychosis itself. Psychosis can result in post-traumatic growth and other positive changes (Jordan et al., 2018). Our data also suggest that symptomatic remission may be a prerequisite to functional and vocational recovery. Therefore, participants experiencing psychosis at follow-up may have struggled to access aspects of meaning in life derived from social/occupational engagement (Leamy et al., 2011).

DUP rather than DUI was a stronger predictor of all scale outcome measures (personal recovery [RAS] and resilience [CD-RISC]) as well as the categorical outcomes of PANSS remission and full personal recovery (PRQ). Our findings deemphasise the role of the length of prodrome in FEP outcome and contribute to the debate on whether or not DUP is an epiphenomenon of premorbid functioning. Our analysis controlled for the potential influence of premorbid social adjustment and concluded that DUP independently impacted outcome at 20 years. The efficacy of initiatives to reduce DUP is equivocal (Oliver et al., 2018). While intensive public awareness campaigns have the potential to decrease DUP (Lloyd-Evans et al., 2011), emphasising biogenetic causal explanations of psychosis in such campaigns may unintentionally enhance stigma increasing DUP (O’Keeffe et al., 2016). These campaigns may also benefit from targeting socially fragmented neighbourhoods (O’Donoghue et al., 2016). The widespread egalitarian rollout of early intervention services and/or integrating psychosis specific pathways to care into front-line services may help reduce delays to treatment. The concept of ‘structural violence’ may similarly play a role. This refers to how social structure can perpetuate inequity, causing preventable suffering by impairing fundamental human needs. Unequal life opportunities impact the likelihood of positive outcomes. For example, people of low socio-economic status tend to have a longer DUP and present to services at an earlier age (Kelly, 2005).

Our finding that older age at first presentation predicted resilience (CD-RISC) is intuitive as people are more likely to have established markers of resilience (e.g. demonstrating personal competence, forming secure relationships, experiencing stress as strengthening) the longer they have lived prior to experiencing psychosis. Older people may possess more resilience supports to buffer against the adverse effects of a FEP which could translate to greater resilience in later life. This result corresponds to research which has found that age at time of exposure to a traumatic event is significantly positively correlated with post-traumatic growth (Meyerson et al., 2011). This relationship may also be mediated by the impact of adolescent onset FEP on social disability, detracting from resilience.

We found that receiving a non-affective diagnosis at baseline predicted personal recovery (RAS). Evidence suggests that people diagnosed with affective psychosis exhibits better outcomes in terms of hospitalisation, remission, social contact and employment (Singh et al., 2000). It may be that personal recovery is not strongly influenced by these variables. Findings may also be explained by diagnostic shifting. Diagnostic uncertainty is frequently a feature of FEP due to the often fluctuating nature of affective and psychotic symptoms evident during the episode (Kim et al., 2011). We used baseline SCID-IV diagnosis in our models. Cohort members may have had their diagnosis revised when they stabilised at later time points shifting between affective and non-affective categories. Taken at face value however, our data point to a possible compounding effect of the combination of affective and psychotic symptomatology on personal recovery.

Exhibiting poor premorbid social adjustment was found to be a significant predictor of all categorical outcome variables measured: FFR, PANSS Remission and PRQ (Fully Recovered). The ability of premorbid adjustment to predict functional outcome and relapse in FEP is widely documented (Alvarez-Jimenez et al., 2012; Santesteban-Echarri et al., 2017). Our findings suggest that functional disability occurs prior to the onset of a FEP and subsequently impacts clinical and personal recovery much later in life. Further prodrome research will indicate whether or not the limited resources of early intervention services should be used to target the first stages of social disability, rather than awaiting the onset of a FEP.

Considering that ‘vocational functioning’ and ‘achievement in role adopted’ are criteria for FFR, it is unsurprising that being in full-time employment at baseline independently predicted FFR. Having a job to provide structure, independence and meaning during/after FEP treatment may buffer against later life deterioration in symptomatology and functioning. In schizophrenia, baseline employment status has been shown to predict time to relapse (Trapp et al., 2013) and employment may in its own right safeguard against hospitalisation (Luciano et al., 2016). Therefore, people who are in employment at FEP onset may be less exposed to trauma in their recovery journey as a consequence.

Counterintuitively, we found that having a lower baseline GAF score increased the likelihood of being in FFR at 20 years. This may reflect the acute nature of clinical presentation at first contact and the severe impact of a once off psychotic episode which resolves itself quickly (e.g. a substance-induced FEP). Acute presentation is typically associated with better prognosis due to prompt identification of treatment needs and enhanced treatment response (Marshall et al., 2005). Findings may also be explained by problems with the psychometric reliability, concurrent validity and predictive utility of the GAF. Not living alone at baseline predicted FFR at 20 years. This effect may relate to the absence of someone to engage in reality testing with, in the home environment; social support from partners, friends, family and housemates; and the role of these cohabitants in facilitating help-seeking and reducing relapse. Supported socialisation and efforts to address romantic loneliness in people with experience of FEP may afford novel routes to cohabiting.

### Strengths and limitations

Study strengths include its epidemiological conception and purpose, its utilisation of an incidence cohort and prospective design, its duration of follow-up and the use of a highly reliable instrument (SCID-IV) to diagnose FEP at baseline. We achieved an acceptable recruitment rate (considering our follow-up length) and we successfully traced 87.1% of the original cohort. Our ability to comprehensively compare very long-term outcomes in FEP and examine their retrospective predictors is unparalleled in the literature. We found no other FEP outcome study that asked participants to assess their recovery according to their own conceptualisation.

Findings should be interpreted with caution due to a number of limitations. While over half of the living cohort members participated and there were no significant differences in baseline characteristics between those followed up or not, it is difficult to generalise the results beyond those who participated. We did not conduct a leakage study to identify cases potentially missed by our screening procedure. It is possible that some people, who developed a FEP within our catchment during the stated timeframe, received treatment elsewhere and thus were not included in our cohort. No reasons were given by individuals for refusing to participate – it may, for example, indicate a desire to move on from a FEP 20 years in the past which is no longer relevant to the individual or may indicate current severity of symptoms impacting participation. Missing data due to attrition are unlikely to be missing at random which limits the use of multiple imputation methods and there is no agreement in the literature on how to handle missing data caused by death during follow-up (Biering et al., 2015). We have, however, estimated recovery rates in the full cohort using different outcome assumptions in those not followed up and those that died. While we were guided by the literature and available baseline data on which variables to explore in model building, there may be other unmeasured variables which are important predictors of the outcomes measured. For example, we did not collect data on mode of onset. Therefore, we were unable to control for this potential confounding factor. As insidious onset predicts poorer outcome in FEP (Chang et al., 2012) and insidious onset and longer DUP are related (Morgan et al., 2006) – the association between DUP and outcome we found may be confounded by mode of onset. No information regarding treatments received or cohort members’ degree of adherence to them was included in our analysis. Consequently, it is impossible to determine if interventions provided impacted recovery/resilience trajectories. The use of statistical methods for repeated correlated measurements (such as generalised estimating equations) may help us understand – not just what helps increase the likelihood of positive outcome in FEP – but at what time point are these factors the most efficacious. Our sample size and analytic strategy required us to dichotomise diagnosis, preventing us examining the effects of individual diagnostic categories. Finally, the relatively small sample size and number of events (remission, personal and clinical recovery) also resulted in uncertainty in the parameter estimates, with wide confidence intervals.

## Conclusion

In the analysis detailed in this article, we sought to conceptualise outcome in FEP in line with contemporary developments in mental health policy/practice and to rigorously investigate the differing impact of baseline predictors on each outcome. In doing so, we found that full remission of psychotic symptoms and personally defined recovery among participants was not just possible but likely in the very long term. However, we concluded that attaining positive functional outcomes (i.e. social relationships, valued roles/occupation and living task performance) and building resilience in FEP remain key challenges for mental health services. Addressing these deficits must be a priority in the Functional Recovery Era.
